# The key clock component ZEITLUPE (ZTL) negatively regulates ABA signaling by degradation of CHLH in Arabidopsis

**DOI:** 10.3389/fpls.2022.995907

**Published:** 2022-09-13

**Authors:** Yongtao Yu, Sergi Portolés, Yi Ren, Guangyu Sun, Xiao-Fang Wang, Huihui Zhang, Shaogui Guo

**Affiliations:** ^1^National Watermelon and Melon Improvement Center, Beijing Academy of Agriculture and Forestry Sciences, Key Laboratory of Biology and Genetic Improvement of Horticultural Crops (North China), Beijing Key Laboratory of Vegetable Germplasm Improvement, Beijing, China; ^2^MOE Key Lab of Bioinformatics, Center for Plant Biology, School of Life Sciences, Tsinghua University, Beijing, China; ^3^Key Laboratory of Saline-alkali Vegetation Ecology Restoration, Ministry of Education, College of Life Sciences, Northeast Forestry University, Harbin, Heilongjiang, China

**Keywords:** ABA, circadian clock, ZTL, CHLH, Arabidopsis, signal transduction

## Abstract

Ubiquitination-mediated protein degradation plays important roles in ABA signal transduction and delivering responses to chloroplast stress signals in plants, but additional E3 ligases of protein ubiquitination remain to be identified to understand the complex signaling network. Here we reported that ZEITLUPE (ZTL), an F-box protein, negatively regulates abscisic acid (ABA) signaling during ABA-inhibited early seedling growth and ABA-induced stomatal closure in *Arabidopsis thaliana*. Using molecular biology and biochemistry approaches, we demonstrated that ZTL interacts with and ubiquitinates its substrate, CHLH/ABAR (Mg-chelatase H subunit/putative ABA receptor), to modulate CHLH stability via the 26S proteasome pathway. CHLH acts genetically downstream of ZTL in ABA and drought stress signaling. Interestingly, ABA conversely induces ZTL phosphorylation, and high levels of ABA also induce CHLH proteasomal degradation, implying that phosphorylated ZTL protein may enhance the affinity to CHLH, leading to the increased degradation of CHLH after ABA treatment. Taken together, our results revealed a possible mechanism of reciprocal regulation between ABA signaling and the circadian clock, which is thought to be essential for plant fitness and survival.

## Introduction

The phytohormone abscisic acid (ABA) regulates many developmental processes including embryo maturation, seed germination, seedling growth, fruit ripening and senescence, and is a vital hormone in plant adaptation to adverse conditions such as drought, salt and cold stresses (Finkelstein et al., 2002; Hunter et al., 2004; Adie et al., 2007; Cutler et al., 2010; Jia et al., 2011b). Over the past two decades, much progress has been made toward understanding the intricate ABA signaling network, and numerous ABA signaling components, including receptors and E3 ligases for ABA, have been identified (Finkelstein et al., 2002; Gao et al., 2007; Johnston et al., 2007; Liu et al., 2007a,b; Guo et al., 2008; Ma et al., 2009; Pandey et al., 2009; Park et al., 2009; Santiago et al., 2009; Cutler et al., 2010; Jaffé et al., 2012; Bueso et al., 2014; Irigoyen et al., 2014; Yang et al., 2016; Yu et al., 2016; Chen et al., 2021). The START-domain superfamily PYR/PYL/RCAR proteins are the best characterized as cytosolic ABA receptors by directly inhibiting type 2C protein phosphatases, then leading to the activation of the SNF1-related protein kinases SnRK2s, which subsequently phosphorylate transcription factors such as ABA response element binding factors (ABFs), to regulate ABA responsive gene expression (Ma et al., 2009; Park et al., 2009; Santiago et al., 2009; Cutler et al., 2010; Gong, 2021).

Chloroplasts are responsible for the bulk of terrestrial photosynthetic primary production, and play vital roles in plant adaptation to both biotic and abiotic stresses (Lopez-Juez and Pyke, 2005; Richardson et al., 2017; Jarvis, 2019). Chloroplast development depends on the import of thousands of nucleus-encoded proteins from the cytosol, and import of these proteins is initiated by TOC (translocon at the outer envelope of chloroplasts) complexes, which are localized in the outer envelope membrane (OEM) of chloroplasts (Li and Chiu, 2010; Ling et al., 2012; Demarsy et al., 2014). Proteolytic regulation of specific chloroplast proteins plays important roles in maintaining normal organellar functions and in delivering responses to developmental and environmental cues (Jarvis and López-Juez, 2013; Richardson et al., 2017). Previous studies have shown that the components of protein import translocases are targeted by the OEM-localized E3 ubiquitin ligase SP1 and subsequently degraded by the cytosolic 26S proteasome. Further studies found that SP2 cooperates with Cell-division-cycle protein 48 (CDC48) to bring about retrotranslocation of ubiquitinated substrates from the OEM, and then enable degradation of the substrates by the cytosolic 26S proteasome (Ling et al., 2012, 2019). However, the mechanisms underlying the ubiquitin-dependent degradation of chloroplast-associated proteins are still largely unknown.

The chloroplast magnesium protoporphyrin IX chelatase large subunit (Mg-chelatase H subunit CHLH/putative ABA receptor ABAR) was reported to function as a candidate receptor for ABA in *Arabidopsis thaliana* (Shen et al., 2006; Wu et al., 2009; Du et al., 2012). Further studies showed that CHLH/ABAR regulates a complicated ABA signaling pathway, in which a group of WRKY transcription factors (WRKY40, WRKY18 and WRKY60), the cochaperonin CPN20, and the PPR protein SOAR1 are involved (Shen et al., 2006; Wu et al., 2009; Shang et al., 2010; Du et al., 2012; Liu et al., 2012; Yan et al., 2013; Zhang et al., 2013, 2014; Jiang et al., 2014, 2015; Mei et al., 2014; Bi et al., 2019; Ma et al., 2020). Although it is still controversial whether CHLH/ABAR binds ABA (Shen et al., 2006; Müller and Hansson, 2009; Wu et al., 2009; Tsuzuki et al., 2011; Du et al., 2012), it has been well confirmed that CHLH/ABAR positively affects ABA signaling (Legnaioli et al., 2009; Tsuzuki et al., 2011). Interestingly, it has been demonstrated that CHLH/ABAR mediates ABA signaling in fruit ripening of both peach (Jia et al., 2011a) and strawberry (*Fragaria ananassa*; Jia et al., 2011b). Taken together, the above results support that CHLH/ABAR has an essential role in ABA signaling.

ZTL belongs to a small, unique family of F-box proteins [ZTL, FLAVIN-BINDING KELCH REPEAT F-BOX1 (FKF1) and LOV KELCH PROTEIN2 (LKP2)] containing an N-terminal LOV (light, oxygen or voltage sensing) domain, an F-box domain and a C-terminus with six Kelch repeats (Demarsy and Fankhauser, 2009; Ito et al., 2012). Previous studies showed that ZTL specifically interacts with the timing of CAB expression 1 (TOC1) and PSEUDORESPONSE REGULATOR 5 (PRR5), and mediates TOC1 and PRR5 proteasomal degradation by an SCF*^ZTL^* complex, which is thought to be important for regulating clock function (Más et al., 2003; Han et al., 2004; Yasuhara et al., 2004; Kevei et al., 2006; Kiba et al., 2007; Baudry et al., 2010). A recent study also showed that ZTL and PRR5 interact with Open Stomata 1 (OST1)to regulate ABA-induced stomatal closure (Jurca et al., 2022). Moreover, TOC1 was reported to be involved in ABA-induced stomatal closure and plant responses to drought by repressing the circadian expression of *CHLH* (Legnaioli et al., 2009).

Many ABA-related processes have been shown to be regulated by the circadian clock in plants, including stomatal movements (Harmer et al., 2000; Dodd et al., 2005; Seung et al., 2012), osmotic and cold responses (Bieniawska et al., 2008; Matsui et al., 2008), hormone signaling and metabolism (Covington et al., 2008), calcium ion fluxes (Johnson et al., 1995) and cyclic adenosine diphosphate ribose (cADPR; Dodd et al., 2007). Additionally, the functional clustering of clock microarray datasets revealed that the key enzymes involved in ABA biosynthesis such as *NINE-CIS-EPOXYCAROTENOID DIOXYGENASE* (*NCED3*) and *ABA DEFICIENT 2* (*ABA2*), and components of ABA signaling or stress response pathways such as *EARLY RESPONSE TO DEHYDRATION 7*, *10*, *12* (*ERD7, ERD10* and *ERD12*), *COLD REGULATED 15 A* (*COR15A*) and *B* (*COR15B*), and *RESPONSE TO DESICCATION* (*RD29A*) are circadian clock-controlled (Covington et al., 2008; Mizuno and Yamashino, 2008). Furthermore, ABA levels show circadian rhythms, and the clock is able to modulate plant sensitivity to ABA (Robertson et al., 2009; Seung et al., 2012). Conversely, application of ABA also lengthens circadian periodicity, and the effect is likely dependent on light, suggesting that ABA can function in the circadian system (Hanano et al., 2006).

Although transcriptome analysis and other studies have indicated an important role of the circadian clock in modulating ABA-mediated responses (Covington et al., 2008; Kidokoro et al., 2009, 2021; Legnaioli et al., 2009), the molecular mechanisms underlying how ABA regulates the circadian clock and the crosstalk between ABA signaling and the circadian clock are poorly defined. Here, we reported that the E3 ubiquitin ligase ZTL negatively regulates ABA signaling by promoting proteasome-mediated degradation of CHLH. These results provide a vital molecular mechanism to explain how the circadian clock regulates ABA signaling. Additionally, we found that high levels of ABA induce ZTL protein phosphorylation. This is a possible molecular mechanism of how ABA signaling modulates the circadian clock. Taken together, our results reveal a possiblemolecular mechanism of bidirectional regulation between ABA signaling and the circadian clock at the post-translational level, which is thought to be crucial for plant fitness and survival.

## Materials and methods

### Plant materials and growth conditions

*Arabidopsis thaliana* seeds were stratified at 4°C in the dark for 3 days on Murashige and Skoog (MS) agar medium supplemented with 3% sucrose and then transferred to a growth chamber at 19–20°C with ∼80 μmol m^–2^ s^–1^ of cool white fluorescent light or in compost soil with ∼120 μmol photons m^–2^ s^–1^ under long day conditions (LgD, 16 h light: 8 h dark).

To generate transgenic plant lines overexpressing the *ZTL* gene, the open reading frame (ORF) for the *ZTL* (At5g57360) was first ligated into pDONR221, and then cloned into pEarleyGate101 by the Gateway method (Earley et al., 2006). Primers used in vector construction are listed in Supplementary Table 1. The construct was verified by sequencing and introduced into the GV3101 strain of *Agrobacterium tumefaciens*. The construction was transformed by floral infiltration into WT (C24) plants. Transgenic plants were selected by basta resistance and confirmed by PCR. All positive lines showed a similar and reported phenotype: long-hypocotyl compared with wild-type (WT, C24) plants (Somers et al., 2004), suggesting these transgenic lines are *ZTL*-overexpressing plants, and the homozygous T3 seeds of the transgenic plants were used for further analysis. Mutant plants *ztl-1* (C24; Somers et al., 2000) and *ztl-3* (Col; Jarillo et al., 2001) were described in the corresponding published papers.

### Phenotypic analysis

To assay germination, approximately 100 seeds were sterilized and planted on MS medium containing 3% sucrose and 0.8% agar (pH5.9), and was supplemented with or without different concentrations of (±) ABA. Germination was defined as an obvious emergence of the radicle through the seed coat. Seedling growth was also assessed by directly planting the seeds in ABA-free MS media and ABA-containing MS medium. For stomatal aperture assays, 3–4-week-old seedlings were used to study ABA-induced stomatal closure, leaves were detached at Zeitgeber Time 2 (ZT2) and floated in the buffer containing 50 mM KCl and 10 mM MES-KOH (pH 6.15) under a halogen cold-light source (Colo-Parmer) at 200 μmol m^–2^ s^–1^ for 3 h followed by addition of 20 μM (±) ABA. Stomatal apertures were recorded on epidermal strips before ABA treatment and 1 h or 3 h after ABA treatment. For water-loss assays, rosette leaves of comparable size from 3 to 4-week-old plants grown under LgD were detached, placed on a Petri dish and weighed at different times after detachment. For drought tolerance test, approximately 8-day-old seedlings were transplanted to the soil (each pot contained 4 plants) for another 2 weeks under standard growth conditions. Then, the plants were subjected to progressive drought by withholding water for 2 weeks and then rewatered for 2 days. The entire test was repeated five times.

### Quantitative PCR

Total RNA was isolated from 14-day-old seedlings with a Total RNA Rapid Extraction Kit (BioTeke, China), and treated with RNase-free DNase I (NEB) at 37°C for 1 h to degrade genomic DNA. Then RNA was purified by a RNA Purification Kit (BioTeke, China). Two micrograms of total RNA was subjected to first-strand cDNA synthesis using a Roche Transcriptor First Strand cDNA Synthesis Kit and an oligo (dT18) primer. qPCR was performed with SYBR Premix Ex Taq (Takara) using a BioRad Real-Time System CFX96TM C1000 Thermal Cycler (BioRad). Amplification of *ACTIN2*/*8* genes was used as an internal control. The primers of *CHLH* and *ACTIN2/8* for qPCR were described previously (Shang et al., 2010). For all quantitative real-time PCR analyses, the assays were repeated three times, and the means of four biological experiments were used to estimate gene expression.

### Western-blot assays

Western-blot assays were essentially performed as previously described (Shen et al., 2006; Wu et al., 2009). Briefly, 2-week-old seedlings were ground in liquid nitrogen and proteins were extracted in extraction buffer [50 mM Tris–HCl, pH 7.4, 150 mM NaCl, 1 mM EDTA, 0.1% (v/v) Triton X-100, 10% (v/v) glycerol and protease inhibitor cocktail (Roche)]. Protein concentration was calculated using the Bradford method (Bio-Rad), and 20–60 μg of total protein was loaded per lane. Proteins were transferred to nitrocellulose membranes (Amersham) and stained with red ponceae following standard protocols. Anti-CHLH (Wu et al., 2009), anti-ACTIN (Shang et al., 2010), anti-GFP (ZA009, Ythx Biotechnology), anti-ubiquitin (SC-166553, Santa Cruz Biotechnology) and anti-cLUC antibodies (Sigma, Cat No. L2164) were used to detect CHLH, ACTIN, ZTL-YFP, polyubiquitinated CHLH proteins and ZTL-cLUC, respectively. For hormone treatment, seedlings were sprayed with 7 ml of 0.005% Triton X-100 aqueous solution containing 100 μM (±) ABA, and samples were collected at the indicated time points.

### Yeast two-hybrid assay

The Matchmaker Gal4 two-hybrid system (Clontech, Cambridge, MA, United States) was used for screening the *Arabidopsis* cDNA library (ABRC, CD4-22). The ORFs of the N-terminus (amino acids 1–691), the middle fragment (amino acids 348–1,038) and the C-terminus (amino acids 692–1,381) of CHLH were fused to the GAL4 DNA binding domain in the plasmid pGBKT7 and were previously described (Shang et al., 2010; Zhang et al., 2014). The ORF of ZTL was cloned into pGADT7 plasmid by the *Nde*I (5’-end) and *Xho*I (3’-end) sites. Primers used in the vector construction are listed in Supplementary Table 1. Different combinations of plasmids were transformed into the yeast strain AH109. Transformants were plated on Leu-Trp-deficient and Leu-Trp-His-Ade-deficient medium separately and grew for 5–7 days at 30°C. pGBKT7-p53 and pGADT7-T were used as a positive control.

### Luciferase complementation imaging

Firefly luciferase complementation imaging (LCI) assay was performed as previously described (Chen et al., 2008). The ORF of ZTL was inserted into cLUC at *Kpn*I and *Sal*I sites. Primers used in the vector construction are listed in Supplementary Table 1. The CHLH-nLUC construct was previously described (Shang et al., 2010). The constructs were transformed into *A. tumefaciens* strain GV3101. Bacterial suspensions were infiltrated into the leaves of 7-week-old Nicotiana benthamiana plants. After infiltration, plants were placed in dark for 44 h, followed by infiltration of an aqueous solution containing 100 μM (±) ABA and placed 4 h in dark again. LUC activity was recorded with a CCD imaging apparatus (Andor iXon).

### Protein purification, pull down and co-immunoprecipitation assays

The full-length cDNA of CHLH and ZTL was ligated into modified pMCSG7 protein expression vectors that contained GST or MBP tags at N-terminus, respectively. Primers used in the vector construction are listed in Supplementary Table 1. Protein purification, pull down and co-immunoprecipitation assays were conducted as previously described (Shang et al., 2010; Zhang et al., 2013).

### CHLH protein degradation assays

For cell-free degradation assays, seedlings were sprayed with 7 ml of 0.005% Triton X-100 aqueous solution containing 100 μM (±) ABA for 2 h before sampling. Samples were ground in liquid nitrogen, resuspended in cell-free buffer (50 mM Tris–HCl pH 7.5, 100 mM NaCl, 5 mM MgCl_2_, 5 mM DTT, 5 μM ATP) and clarified by centrifugation at 3,300 *g*. Equal amounts of extracts were transferred to individual tubes and incubated at 30°C with or without 50 μM MG132 as indicated. Reactions were stopped by adding protein gel-loading buffer. For *in planta* degradation assay, 2-week-old seedlings were transferred to MS liquid medium for 24 h, and then seedlings were incubated with 100 μM cycloheximide with or without 50 μM MG132 for 2 h in agitation, followed by the addition of (±) ABA to a final concentration of 100 μM. Then, samples were collected at the indicated time points and processed following the standard protocol.

### Phosphorylation assays (phos-tag)

For phosphorylation assays, proteins were extracted in AP buffer (10 mM Tris–HCl pH 8.0, 5 mM MgCl_2_, 100 mM KCl, 0.02% Triton X-100) and separated by a Mn^2+^-Phos-tag (Wako) SDS-PAGE following manufacturer’s recommendations. Briefly, standard SDS-acrylamide gels were supplemented with or without 30 μM Phos-tag and 0.2 mM MnCl_2_. Before transferring to nitrocellulose membranes, gels were washed two times with protein transfer buffer (25 mM Tris, 192 mM glycine, 20% (v/v) methanol) supplemented with 1 mM EDTA for 10 min, followed by an additional wash with transfer buffer without EDTA. Subsequent steps were performed according to standard western blot protocols. For alkaline phosphatase treatments, proteins were extracted in AP buffer and incubated with 10 U of FastAP alkaline phosphatase (Thermo Scientific) for 1 h at 30°C.

### Statistical analysis

Statistical analyses were performed using the GraphPad Prism software (GraphPad Software, Inc. United States). For stomatal aperture and plant survival statistical analysis, two-way ANOVA tests followed by Dunnett’s multiple comparison tests were performed. Genotype and time after ABA addition or genotype and plant survival were considered as variables.

## Results

### ZEITLUPE interacts with CHLH

**FIGURE 1:**
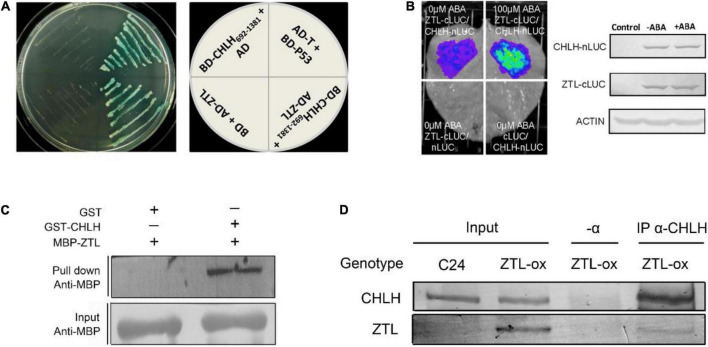
Molecular interaction between CHLH and ZTL. **(A)** CHLH interacts with ZTL in yeast. The C-terminus (amino acids 692–1,381) of CHLH was fused to GAL4 DNA binding domain in the plasmid pGBKT7 (BD-CHLH_692–1,381_), and the ORF of ZTL was cloned into pGADT7 plasmid (AD-ZTL). BD-p53/AD-T was taken as a positive control. AD/BD-CHLH_692–1,381_ and AD-ZTL/BD were used as negative controls. The experiments were repeated three times with similar results. **(B)** Firefly luciferase complementation imaging (LCI) assay. CHLH was fused with nLUC (CHLH-nLUC), and ZTL was fused with cLUC (ZTL-cLUC). Tobacco leaves were transformed and treated with 100 μM (±) ABA for 4 h prior to image recording. ZTL-cLUC/nLUC and cLUC/CHLH-nLUC were used as negative controls. The protein amounts of CHLH-nLUC and ZTL-cLUC in the tobacco leaves with or without ABA treatment described in the experimental group (CHLH-nLUC/ZTL-cLUC), which were detected by immunoblotting using anti-CHLH and anti-cLUC antiserum, respectively. The expression of ACTIN protein was used as an internal control. “Control” indicates leaves without transformation. The experiments were repeated four times with similar results. **(C)** Pull-down assay showed that CHLH directly interacts with ZTL. The experiments were repeated three times with similar results. **(D)** Western-blot analysis of protein extracts immunoprecipitated with anti-CHLH antibody (α-CHLH) and subsequent detection of ZTL-YFP (α-GFP) protein. The experiments were repeated three times with similar results.

CHLH was reported to regulate a vital central ABA signaling network (Shang et al., 2010; Zhang et al., 2013, 2014; Mei et al., 2014), and the cytosolic C-terminus of CHLH plays a central role in ABA signaling (Wu et al., 2009). In a yeast two-hybrid screen using the C-terminus of CHLH (amino acids 692–1,381) as a bait, and from 3 × 10^6^ colonies screened, we found a CHLH interaction partner that is a clock-associated PAS protein, ZTL. Moreover, we conducted IP-MS assays to screen interaction partners of ZTL *in vivo* using an anti-GFP antibody in ZTL-overexpressing plants (with a YFP tag at the C-terminus of ZTL, named ZTL-ox). Our MS results confirmed previously reported results that TOC1, PRR5 and GIGANTEA (GI) interact with ZTL, and showed that CHLH was associated with ZTL *in vivo* (Supplementary Data 1, 2). Furthermore, we performed yeast two-hybrid, pull-down, firefly luciferase complementation imaging (LCI) and co-immunoprecipitation (Co-IP) assays to confirm the interaction *in vitro* and *in vivo*. In the yeast two-hybrid system, the C-terminus of CHLH_692–1,381_ interacts with ZTL (Figure 1A), indicating that the CHLH-ZTL interaction takes place in the C-terminal cytosolic portion of CHLH. Then, we tested whether other truncated CHLHs interact with ZTL. Our results showed that the N-terminus of CHLH_1–691_ (Shang et al., 2010) does not interact with ZTL (Supplementary Figure 2C), while the central region of CHLH_348–1,038_ (Zhang et al., 2014) interacts with ZTL in yeast (Supplementary Figure 2A), suggesting that the interaction region of CHLH and ZTL may be from the C-terminal amino acids 692–1,038 of CHLH (Supplementary Figure 2B). Moreover, our pull-down assays indicated that ZTL directly interacts with CHLH *in vitro* (Figure 1C). Furthermore, we confirmed the CHLH-ZTL interaction *in vivo* with LCI and Co-IP assays (Figures 1B,D), and found that ABA enhances the physical interaction between CHLH and ZTL in tobacco (Figure 1B).

### ABA-mediated responses are altered in *ZEITLUPE* miss-expressing plants

**FIGURE 2:**
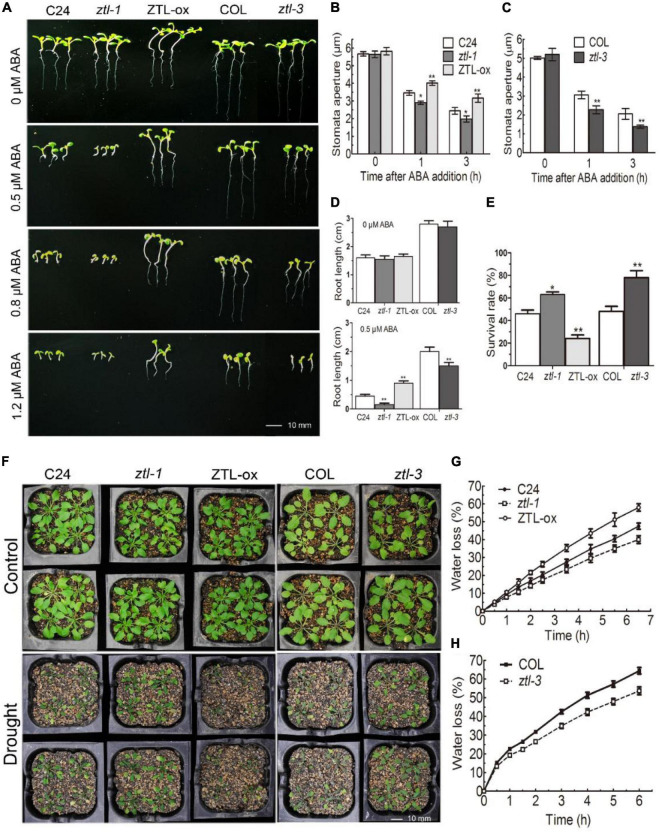
ZTL negatively regulates ABA signaling. **(A)** Comparative photographs of ten-day-old C24, *ztl-1* (C24), ZTL-ox (C24), Col and *ztl-3* (Col), seedlings grown on MS plates containing 0, 0.5, 0.8 or 1.2 μM (±) ABA. **(B,C)** Stomatal apertures of C24, *ztl-1*, ZTL-ox **(B)**, Col and *ztl-3* **(C)**). Stomatal apertures were measured before ABA treatment and 1 or 3 h after ABA treatment. Data are the means ± SE from four independent experiments with 60 apertures per genotype and per treatment (with significant differences at *P < 0.05, **P < 0.01). **(D)** Statistics of the primary root lengths of the plants as described in **(A)**. Student’s *t*-test was used to compare the primary root lengths of each mutant or transgenic line with their corresponding WT plants (with significant differences at **P < 0.01). **(E)** The survival rates of C24, *ztl-1*, ZTL-ox, Col and *ztl-3* as mentioned in **(F)**. Plants were subjected to progressive drought by withholding water for 2 weeks, and survival rates were recorded 2 days after the plants were re-watered. Data are the means ± SE of 5 independent experiments with at least 96 plants for each genotype (with significant differences at *P < 0.05, **P < 0.01). **(F)** Whole-plant status in drought tolerance assay for C24, *ztl-1*, ZTL-ox, Col and *ztl-3* plants. Plants were well watered (Control) or drought stressed by withholding water (Drought) for 2 weeks. The experiments were repeated five times with similar results. **(G,H)** Water loss rates during a 6.5/6-h period from the detached leaves of C24, *ztl-1*, ZTL-ox **(G)**, Col and *ztl-3* **(H)** plants. Each value is the mean ± SE of three biological determinations.

Previous studies have reported that TOC1 negatively participates in ABA-induced stomatal closure and plant responses to drought by inhibiting *CHLH* messenger RNA (mRNA) expression (Legnaioli et al., 2009). Then, we tested whether ZTL is involved in ABA signaling correlating with its known role in TOC1 degradation. To that end, we used two background Arabidopsis plants to conduct ABA phenotypic tests: the *ztl-1* mutant (Somers et al., 2000) and ZTL-ox had a C24 background, and the *ztl-3* mutant (Jarillo et al., 2001) had a Col background. In this work, we observed that *ZTL* mutants showed ABA hypersensitive phenotypes in ABA-inhibited early seedling growth and ABA-induced stomatal closure, while *ZTL*-overexpression plants displayed hyposensitive phenotypes (Figures 2A–D). However, *ZTL* miss-expressing seeds did not show significant differences from their corresponding WT seeds in ABA-induced inhibition of seed germination (Supplementary Figure 1).

Drought tolerance phenotypes are commonly associated with enhanced stomatal closure, which can minimize water transpiration from the leaves under drought conditions (Yamaguchi-Shinozaki and Shinozaki, 2006; Sirichandra et al., 2009). Therefore, we checked whether *ZTL* miss-expressing plants exhibited drought tolerance-related phenotypes. Consistent with the results of ABA-induced stomatal closure, *ztl-1* and *ztl-3* plants showed enhanced water-holding capacity and drought tolerance, whereas ZTL-ox plants exhibited reduced capacity to conserve water and drought tolerance (Figures 2E–H). Taken together, our results regarding ABA-induced stomatal closure, water loss and drought tolerance showed that the phenotype of *ZTL* mutants was similar to *TOC1* mutants, and the phenotype of *ZTL*-overexpressing plants was similar to *TOC1-*overexpressing plants, implying that ZTL negatively regulates ABA signaling independent of its role in TOC1 regulation.

### CHLH mRNA levels do not correlate with protein levels in *ZEITLUPE* miss-expressing plants

**FIGURE 3:**
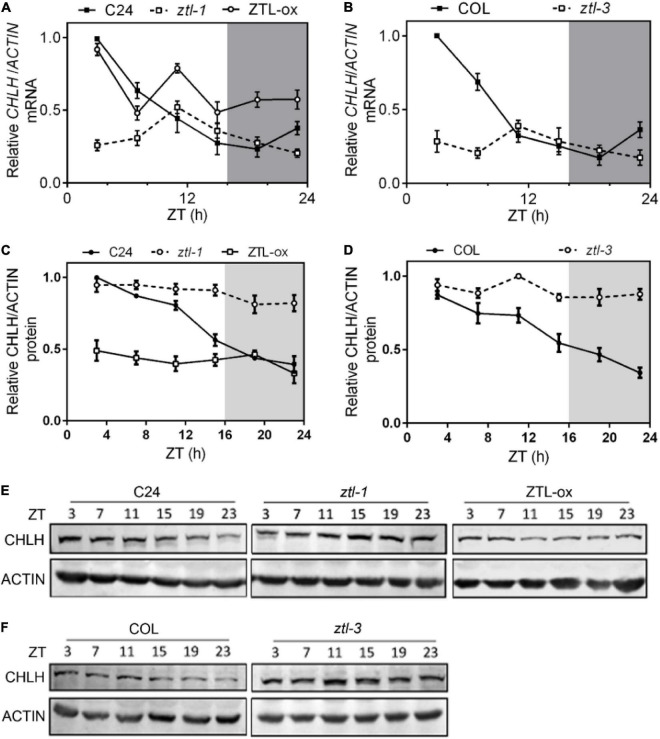
Comparisons of CHLH protein and mRNA abundance in *ZTL* miss-expressing plants. **(A–D)** CHLH mRNA **(A,B)** and protein **(C,D)** expression analysis in C24, *ztl-1*, ZTL-ox, Col and *ztl-3* plants. Seedlings were entrained under long day cycles for 2 weeks, and samples were collected at the indicated Zeitgeber Time (ZT). Means ± SE of four independent experiments are represented relative to the maximum value and normalized to ACTIN expression. **(E,F)** Comparisons of relative CHLH protein abundance in C24, *ztl-1*, ZTL-ox **(E)**, Col and *ztl-3* **(F)** plants by western blot.

Given that ZTL modulates ABA signaling independent of its role in proteasome-mediated degradation of TOC1, we studied whether CHLH mRNA levels correlate with altered TOC1 protein levels in *ZTL* miss-expressing plants. To that end, we performed qPCR assays to determine *CHLH* mRNA levels during the diurnal cycle. Our results showed overall lower transcript levels of *CHLH* in both *ztl-1* (Figure 3A) and *ztl-3* (Figure 3B) plants, whereas a clear up-regulation of *CHLH* was observed in *ZTL*-overexpressing plants (Figure 3A). These results indicated that alteration of *CHLH* RNA levels in *ZTL* miss-expressing plants is most likely due to alterations in TOC1 protein levels, suggesting that ZTL regulates the expression of *CHLH* by TOC1. To obtain a deeper understanding of why altered transcript levels of *CHLH* in *ZTL* miss-expressing plants lead to unexpected phenotypes in ABA-induced stomatal closure and drought tolerance, we studied whether the mRNA and protein levels of CHLH correlate in *ZTL* miss-expressing plants. To that end, we performed western blot analysis to study CHLH protein abundance during the diurnal cycle. Our results showed that CHLH protein displays a clear correlation with mRNA levels in WT (C24 and Col) plants, exhibiting high levels during the first half of the day, gradually decreases during the second half and reaches a minimum before dawn (Figures 3C–F). However, *CHLH* mRNA levels do not correlate with protein levels in *ZTL* miss-expressing plants. *ZTL* mutants displayed overall lower levels of mRNA but higher levels of CHLH protein, and ZTL-ox plants exhibited overall higher levels of mRNA but lower levels of CHLH protein (Figure 3). Higher levels of CHLH protein in *ZTL* mutants and lower levels of CHLH protein in *ZTL*-overexpressing plants are consistent with observed stomatal closure and drought-related phenotypes in *ZTL* miss-expressing plants. Moreover, the diurnal oscillation of CHLH protein was severely disrupted in the *ztl-1* and *ztl-3* mutants (Figures 3C–F). Taken together, our qPCR and western blot results suggested that ZTL might regulate CHLH protein stability at the post-translational level.

### ABA induces CHLH protein degradation via the ubiquitin-dependent 26S proteasome pathway

**FIGURE 4:**
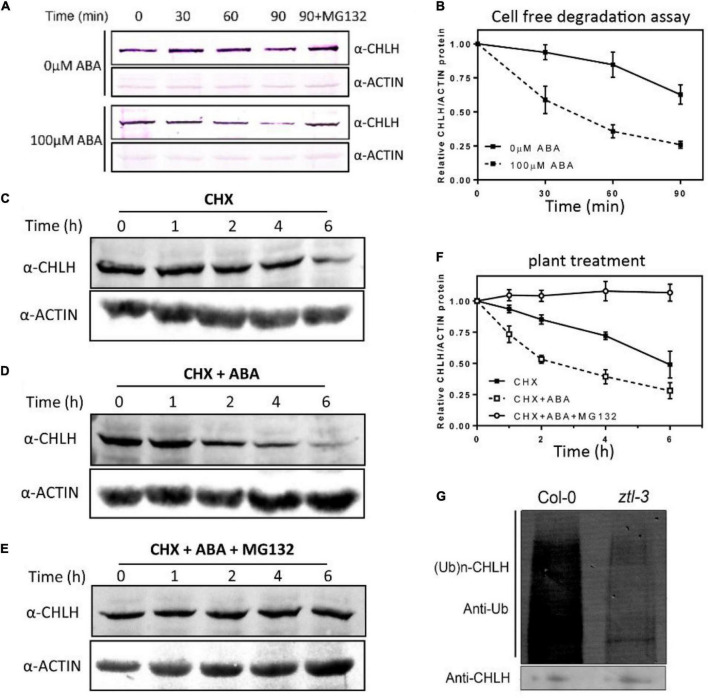
ABA induces CHLH proteasomal degradation. **(A)** Immunodetection of CHLH protein *in vitro*. Seedlings were grown in LgD conditions and treated with or without 100 μM (±) ABA for 2 h prior to sampling. Protein extracts were incubated at 30°C in cell-free buffer with or without proteasome inhibitor (MG132) for the indicated time (min). **(B)** Quantification of CHLH protein abundance in cell-free assays. Data are shown as the means ± SE of three independent experiments relative to the initial value. **(C–E)** Immunodetection of CHLH protein *in vivo*. Seedlings were grown in LgD, and seedlings were treated with cycloheximide (CHX) and with or without proteasomal inhibitor (MG132) for 2 h at ZT3. Then, the seedlings were treated with or without 100 μM (±) ABA. **(F)** Quantification of CHLH protein abundance in *in vivo* degradation assays. Data are shown as the means ± SE of three independent experiments relative to the initial value and normalized to ACTIN abundance. **(G)** Polyubiquitination of CHLH *in vivo*. Total protein extract was immunoprecipitated with anti-CHLH antibody at ZT19, and precipitated proteins were subjected to immunoblot analysis with anti-ubiquitin antibody (upper panel). Immunoblot analysis of the total protein extract using anti-CHLH antibody as a loading control (lower panel). The experiments were repeated three times with similar results.

Our qPCR and western blot results indicate a possible role of ZTL in the post-translational regulation of CHLH (Figure 3). Considering that ZTL is an E3 ligase of the SCF complex, we tested whether CHLH is degraded by the 26S proteasome and whether ABA has any effect on this regulation. Firstly, we examined CHLH protein stability using a previously described cell-free degradation assay (Más et al., 2003). Our results revealed that CHLH protein was unstable and exhibited a subtle reduction over time, and ABA treatment caused a clear reduction in CHLH abundance. However, protein extracts treated with MG132 markedly stabilized CHLH protein regardless of ABA treatment (Figures 4A,B), suggesting that CHLH is degraded by the 26S proteasome pathway, and ABA stimulates the proteasome-dependent degradation of CHLH. To confirm the cell-free results, we performed protein stability assays *in vivo*. *De novo* protein synthesis was first blocked by addition of the protein synthesis inhibitor cycloheximide (CHX). Our studies indicated a clear reduction in CHLH protein over the time course (Figures 4C,F), and this reduction was faster after ABA treatment (Figures 4D,F), while CHLH protein was stable in plants treated with both ABA and MG132 (Figures 4E,F), suggesting that ABA induces CHLH degradation by the 26S proteasome. Moreover, we found that CHLH degradation was repressed in the *ztl-3* mutant, and ABA also did not induce CHLH degradation in the *ztl-3* mutant (Supplementary Figure 3).

Meanwhile, we detected the polyubiquitination of CHLH *in vivo*. Total protein extract was immunoprecipitated with anti-CHLH antibody, and precipitated proteins were subjected to immunoblot analysis with anti-ubiquitin antibody (Figure 4G, upper panel). Our western blot results showed that ubiquitination of CHLH was significantly attenuated in the *ztl-3* mutant at ZT19. Taken together, our results support the notion that ABA induces CHLH degradation via the ubiquitin-dependent 26S proteasome pathway, and ZTL is responsible for CHLH degradation in plants.

### ABA induces ZEITLUPE phosphorylation

Our results indicate that external application of ABA enhances the interaction between ZTL and CHLH and ZTL-mediated CHLH proteasomal degradation (Figures 1B, 4). However, the molecular mechanism underlying how ABA changes the affinity of ZTL to CHLH needs to be explored.

**FIGURE 5:**
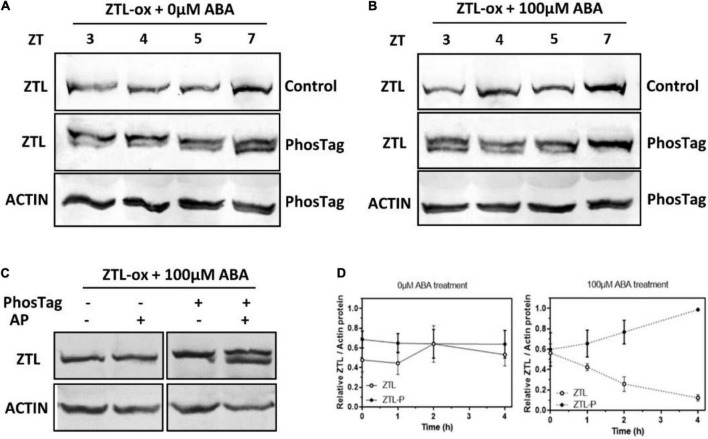
ABA promotes ZTL phosphorylation. **(A,B)** Immunodetection of ZTL protein isoforms with α-GFP antibody in ZTL-ox plants. Seedlings were entrained under LgD cycles, and were treated with **(B)** or without **(A)** 100 μM (±) ABA at ZT3. Then samples were collected at the indicated ZT. Total protein extracts were resolved in SDS-PAGE gels with (middle, down) or without (up) Phos-tag. ACTIN protein was used as a control (down). **(C)** Immunodetection of ZTL isoforms in ZTL-ox seedlings with 4-h 100 μM (±) ABA treatment. Seedlings were entrained under LgD cycles, and were treated with 100 μM (±) ABA at ZT3. Then, samples were collected at ZT7. Protein extracts were incubated at 30°C for 1 h in alkaline phosphatase (AP) buffer with or without alkaline phosphatase. Then protein extracts were resolved in SDS-PAGE gels with (right) or without (left) Phos-tag. The experiments were repeated three times with similar results. **(D)** Quantification of non-phosphorylated and phosphorylated ZTL protein abundances in **(A,B)**. Means ± SE of four independent experiments are represented relative to the maximum value and normalized to ACTIN expression.

Previous studies reported that phosphorylation of the E3 ligase smurf1 switches its substrate preference in support of axon development (Cheng et al., 2011). And protein phosphorylation plays crucial roles in circadian clock and ABA signaling (Sugano et al., 1998; Daniel et al., 2004; Fujiwara et al., 2008; Portolés and Más, 2010; Wang et al., 2010; Kulik et al., 2011; Kusakina and Dodd, 2012; Yang et al., 2017). Therefore, we tested whether ZTL is phosphorylated and whether ABA can impact this process. To that end, we used a compound called Phos-tag ligand, which provides phosphate affinity SDS-PAGE for mobility shift detection of phosphorylated proteins (Komis et al., 2014). Our results showed that conventional western blots only exhibited a unique band for the ZTL-YFP protein, while application of Phos-tag to the SDS gels allowed to resolve two ZTL-YFP bands (Figures 5A,B), indicating that ZTL may have at least two isoforms, one phosphorylated and one not. More importantly, our results showed that the external application of ABA progressively reduced the proportion of the lower band (non-phosphorylated ZTL), and the band completely disappeared after 4-h ABA treatment (Figures 5B,D), suggesting that ABA induces ZTL phosphorylation. To further confirm that the two ZTL-YFP bands observed in the Phos-tag blots correspond to phosphorylated and non-phosphorylated isoforms, we treated the plants with 100 μM (±) ABA for 4 h (ZTL was presumably all phosphorylated), and then we treated protein extracts with alkaline phosphatase to recover its un-phosphorylated state. Indeed, the alkaline phosphatase treated extracts displayed two specific bands (Figure 5C), indicating that alkaline phosphatase partially restored the un-phosphorylated state of the ZTL protein pull.

In summary, our results showed that ABA induces ZTL phosphorylation, ABA enhances the interaction between ZTL and CHLH, and induces ZTL-mediated CHLH proteasomal degradation (Figures 1B, 4, 5). These results suggest that the phosphorylated ZTL protein may exhibit higher affinity to CHLH, resulting in lower levels of CHLH protein after ABA treatment.

### Disruption of CHLH suppresses ABA hypersensitivity of the *ZTL* mutant

**FIGURE 6:**
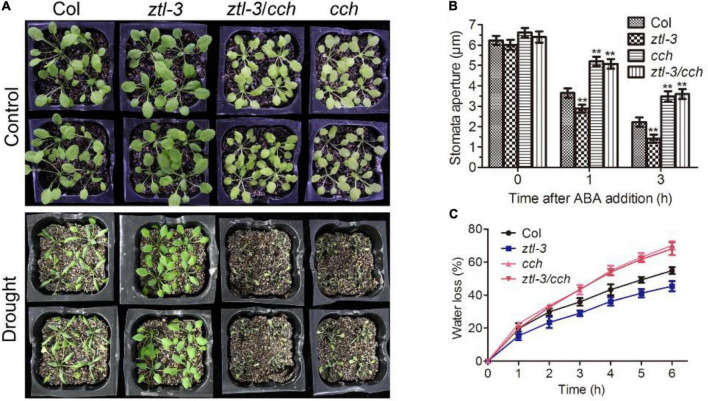
Disruption of CHLH suppresses ABA hypersensitivity of *ztl-3* mutant. **(A)** Whole-plant status in drought tolerance assay for Col, *ztl-3*, *ztl-3*/*cch* and *cch* plants. Plants were well watered (Control) or drought stressed (Drought) by withholding water for 2 weeks or re-watered for 2 d (Re-watered). The experiments were repeated five times with similar results. **(B)** Stomatal apertures of Col, *ztl-3*, *ztl-3*/*cch* and *cch* plants. Stomatal apertures were measured before ABA treatment and 1 or 3 h after ABA treatment. Data are the means ± SE from four independent experiments with 60 apertures per genotype and per treatment (with significant differences at **P < 0.01). **(C)** Water loss rates during a 6-h period from the detached leaves of Col, *ztl-3*, *ztl-3*/*cch* and *cch* plants. Each value is the mean ± SE of three biological determinations.

The *CHLH* mutant *cch* displayed strong insensitivity to ABA in stomatal movement and reduced drought tolerance (Shen et al., 2006; Wu et al., 2009). We introduced the *cch* mutant into the *ztl-3* mutant, and the double mutant *ztl-3 cch* exhibited an extremely similar phenotype to *cch* in ABA-induced stomatal closure, water loss and drought tolerance (Figure 6). These solid genetic data showed that ZTL mediates ABA signaling upstream of CHLH, which is consistent with its role in proteasomal degradation of CHLH.

## Discussion

### ZEITLUPE is involved in ABA signaling independent of its role in TOC1 regulation

The key clock component TOC1 is targeted for proteasome-mediated degradation through specific interaction with ZTL (Más et al., 2003). Previous studies showed that overexpression of *TOC1* led to enhanced abscisic acid sensitivity during seed germination (Castells et al., 2010). Moreover, TOC1 was reported to negatively mediate ABA signaling in ABA-induced stomatal closure and plant responses to drought by inhibition of *CHLH* expression (Legnaioli et al., 2009). Our phenotypic results showed that *ZTL* mutants display enhanced ABA-induced stomatal closure and drought tolerance, similar to *TOC1* mutants, and *ZTL*-overexpressing plants exhibit reduced ABA-induced stomatal closure and drought tolerance, similar to *TOC1*-overexpressing plants (Figure 2). These results indicate that ZTL negatively regulates ABA signaling independent of its role in proteasomal degradation of TOC1. In addition, *ZTL* miss-expressing seeds showed a wild-type ABA response in ABA-inhibited seed germination (Supplementary Figure 1), implying that ZTL may have TOC1-independent roles during germination, which can compensate for its TOC1-dependent defects in ABA-inhibited seed germination. In agreement with this idea, PRR5, another known target of ZTL, was reported to negatively regulate the biosynthetic pathways of ABA (Kiba et al., 2007; Fukushima et al., 2009). Moreover, EARLY BIRD (EBI) was reported to associate with ZTL and regulate abiotic stress signaling and the circadian clock (Lisso et al., 2006; Johansson et al., 2011). A recent study reported that ZTL positively regulates ABA-induced stomatal closure with PRR5 and OST1 under LD (12 h light: 12 h dark) and CO_2_-free aeration (Jurca et al., 2022). Our results showed that ZTL negatively regulates ABA signaling under long day (LgD, 16 h light: 8 h dark) and CO_2_-normal conditions. Due to ZTL is a circadian clock protein, different growth conditions and inconsistent treatment conditions (CO_2_-free aeration/CO_2_-normal conditions) might lead to changes in ZTL interacting proteins at the same sampling time, which might account for the phenotypic inconsistency of ZTL. Taken together, these results suggested that ZTL might mediate complicated ABA signalings in plants. However, our genetic data showed that disruption of *CHLH* suppresses ABA hypersensitivity in the *ztl-3* mutant (Figure 6), supporting the ZTL-CHLH module in ABA signaling. Additionally, our results indicated that ZTL plays an important role in ABA signaling independent of its regulation of TOC1 degradation, and this role is able to overcome its indirect effect on TOC1 protein turnover.

### ZEITLUPE interacts with CHLH and mediates the degradation of CHLH

Our qPCR and western blot results showed that *ZTL* mutants displayed overall lower levels of *CHLH* mRNA but higher levels of CHLH protein, and *ZTL*-overexpression plants exhibited overall up-regulation of *CHLH* mRNA but down-regulation of CHLH protein (Figure 3). These results suggested that alteration of *CHLH* transcript levels in *ZTL* miss-expressing plants correlates with alterations in TOC1 protein levels, indicating that ZTL regulates *CHLH* mRNA expression through proteasomal degradation of TOC1. The inconsistent expression of CHLH mRNA and protein in *ZTL* miss-expressing plants suggests that ZTL may have a role in post-translational regulation of CHLH. Indeed, our genetic and biochemical results showed that CHLH is targeted by ZTL for proteasome-mediated degradation in modulating ABA signaling (Figures 3, 4, 6). CHLH protein exhibited diurnal oscillation in WT plants (Figures 3C–F). This is consistent with the circadian regulation of ZTL protein abundance by GIGANTEA (GI). The GI-ZTL interaction stabilizes the ZTL protein at the end of the day, and then allows higher accumulation of the ZTL protein during the evening (Kim et al., 2007), when CHLH protein abundance is lower (Figures 3C–F).

Turnover of internal chloroplast proteins is controlled by proteases inherited from the organelle’s prokaryotic ancestor, but the molecular mechanisms of ubiquitin-dependent degradation of chloroplast-associated proteins are still poorly understood. Although it is still controversial whether ZTL localizes in the nucleus, it has been well confirmed that ZTL localizes in the cytoplasm (Kim et al., 2007; Takase et al., 2011). CHLH localizes in the chloroplast outer membrane with its N and C termini exposed to the cytosol (Shang et al., 2010). Furthermore, our results in yeast two-hybrid assays indicate that ZTL interacts with the cytosolic C-terminus of CHLH (Figure 1A and Supplementary Figure 2). These results provide a plausible explanation for how CHLH and ZTL proteins can interact at a subcellular level. Previous studies reported that the chloroplast outer envelope membrane (OEM)-localized ubiquitin E3 ligase SP1 is responsible for the degradation of OEM-localized components of protein import translocases via the cytosolic 26S proteasome (Ling et al., 2012, 2019). Further studies found that SP2 and CDC48A cooperated to bring about retrotranslocation of ubiquitinated substrates from the OEM to the cytosol. Interestingly, our IP-MS results also found that CDC48A associates with ZTL *in vivo* (Supplementary Data 1, 2), implying CDC48A may also mediate CHLH retrotranslocation to the cytosol. Taken together, previous and our results together support the conclusion that chloroplast outer envelope membrane proteins are degraded through the cytoplasmic 26S proteasome. However, we first reported that chloroplast outer membrane-localized proteins are targeted by cytosolic ubiquitin E3 ligases for proteasome-mediated degradation, which deepens our understanding of ubiquitin-dependent chloroplast-associated protein degradation in plants.

### Crosstalk between circadian clock and ABA signaling

**FIGURE 7:**
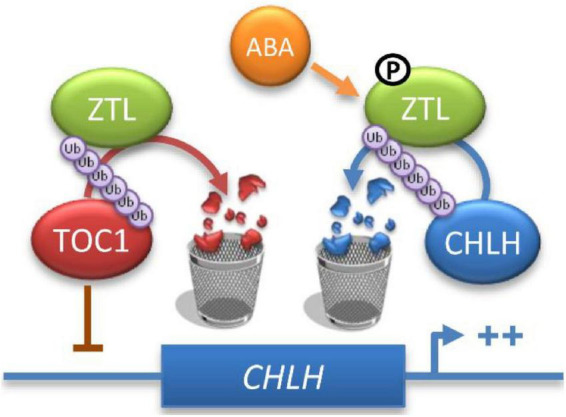
Proposed model for ZTL action in ABA signaling. ZTL may regulate the expression of *CHLH* by TOC1 at the mRNA level. In addition, ZTL also modulates CHLH stability via the 26S proteasome pathway, and ABA enhances CHLH proteasomal degradation. Therefore, ZTL regulates the expression of CHLH at both transcriptional and post-transcriptional levels.

Previous studies revealed that phosphorylation of some clock components affects their subcellular localization, turnover and affinity to their substrates, constituting an important mechanism for clock function in *Arabidopsis*, *Neurospora*, *Drosophila* and humans (Edery et al., 1994; Liu et al., 2000; Bao et al., 2001; Toh et al., 2001; Ko et al., 2002; Akten et al., 2003; Daniel et al., 2004; Merrow et al., 2006; Vanselow and Kramer, 2007; Portolés and Más, 2010; Robles et al., 2017). Our results also indicate that high levels of ABA induce ZTL phosphorylation (Figure 5). Furthermore, we found that ABA enhances ZTL and CHLH interaction (Figure 1B), and induces ZTL-mediated CHLH degradation (Figures 4A–F). These results suggest that phosphorylated ZTL may display a higher affinity for CHLH, resulting in the enhanced degradation of CHLH after ABA treatment. Previous studies showed that ABA-induced *TOC1* expression enhances *TOC1*-mediated transcriptional repression of *CHLH*, while ABA-induced *TOC1* expression also requires a functional CHLH, suggesting that the reciprocal regulation between TOC1 and CHLH might function as a fine-tuned switch that helps to modulate plant sensitivity to ABA, which in turn favors the temporal regulation of plant responses to drought stress (Legnaioli et al., 2009). Consistently, our results showed that ABA represses the expression of CHLH at both transcriptional and post-transcriptional levels. On the one hand, high levels of ABA induce the expression of TOC1 protein by ABA-induced transcriptional regulation of *TOC1* (Legnaioli et al., 2009). On the other hand, ABA stimulates ZTL-mediated CHLH proteasomal degradation, and the “enhanced” repression of CHLH better modulates plant sensitivity to ABA under drought conditions (Figure 7).

In ABA signaling, the RCAR/PYR/PYL-PP2C-SnRK2 module is considered the main pathway, and SnRK2 proteins initiate a phosphorylation cascade in presence of ABA (Ma et al., 2009; Park et al., 2009; Miyakawa et al., 2013). Using the Arabidopsis Interaction Viewer,^1^ we found at least two members of the SnRK2 family of kinases as putative ZTL interactors (Geisler-Lee et al., 2007). Additionally, previous studies reported that SKP1/ASK1, a conserved SCF (Skp1-cullin-F-box) ubiquitin ligase subunit, interacts with SnRK protein kinases to mediate proteasomal binding of a plant SCF ubiquitin ligase, suggesting that SnRK protein kinases are associated with the 26S proteasome in plants (Farrás et al., 2001). Moreover, our IP-MS results showed that SnRK2.1 and SnRK2.2 associate with ZTL *in vivo* (Supplementary Data 1). Taken together, SnRK kinases may be responsible for ZTL phosphorylation in response to ABA. Future studies are needed to confirm this regulation and will be crucial to provide the molecular link between ABA signaling and the circadian clock.

## Data availability statement

The mass spectrometry proteomics data of ZTL IP-MS Repeat 1 (Supplementary Data 1) have been deposited to the ProteomeXchange Consortium^2^ via the iProX partner repository (Ma et al., 2019) with the dataset identifier PXD029110 (Subproject ID: IPX0003606001).

## Author contributions

YY, SG, and X-FW designed the research. YY and SP performed the majority of the experiments and wrote and revised the manuscript with input from all authors. HZ participated in the generation of transgenic plants and the performance of some related ubiquitination assays. YR and GS revised the manuscript and gave good advices for the revised manuscript.

## Footnotes

1
http://bbc.botany.utoronto.ca/interactions/

2
http://proteomecentral.proteomexchange.org
