# Supplementary material for: The key clock component ZEITLUPE (ZTL) negatively regulates ABA signaling by degradation of CHLH in Arabidopsis
Source: Front Plant Sci. 2022 Sep 13;13:995907. doi: 10.3389/fpls.2022.995907 (PMC9513469; doi:10.3389/fpls.2022.995907)
Supplement: Supplementary file 1 [file Data_Sheet_1.docx]

**Supplemental Information**


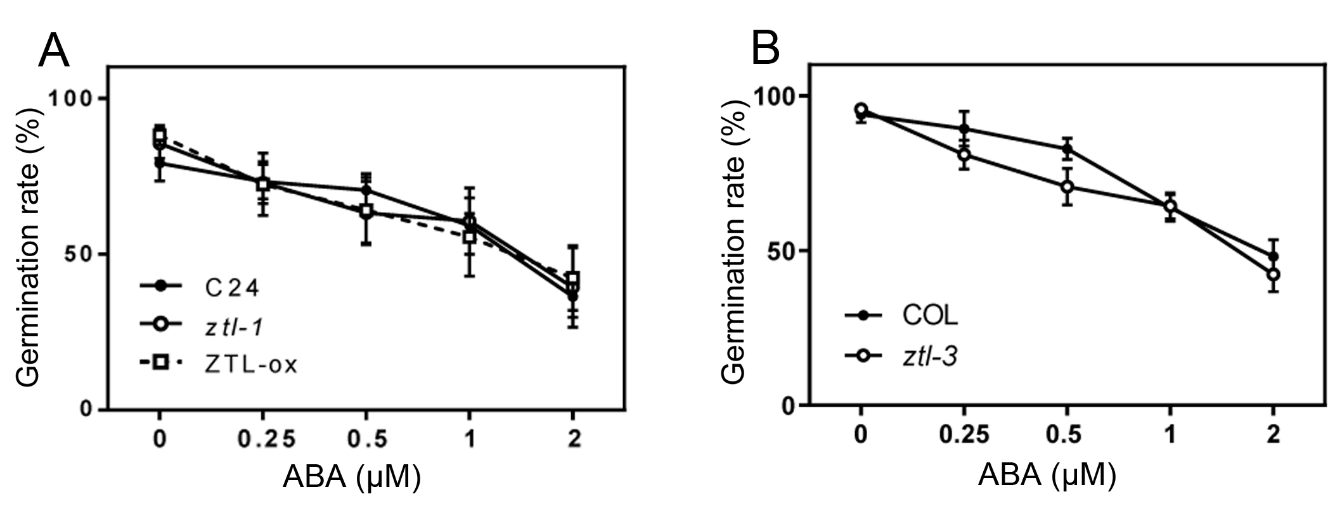


**Supplementary Figure S1**. **Seed germination of *ZTL* miss-expressing plants**

**(A and B)** Germination rates of C24, *ztl-1*, ZTL-ox, Col and *ztl-3*. Seeds were placed on MS plates containing 0, 0.25, 0.5, 1 or 2 μM ABA and the percentage of germinated seeds was scored 48 h after stratification. Data is shown as means ± SE of four independent experiments.

**
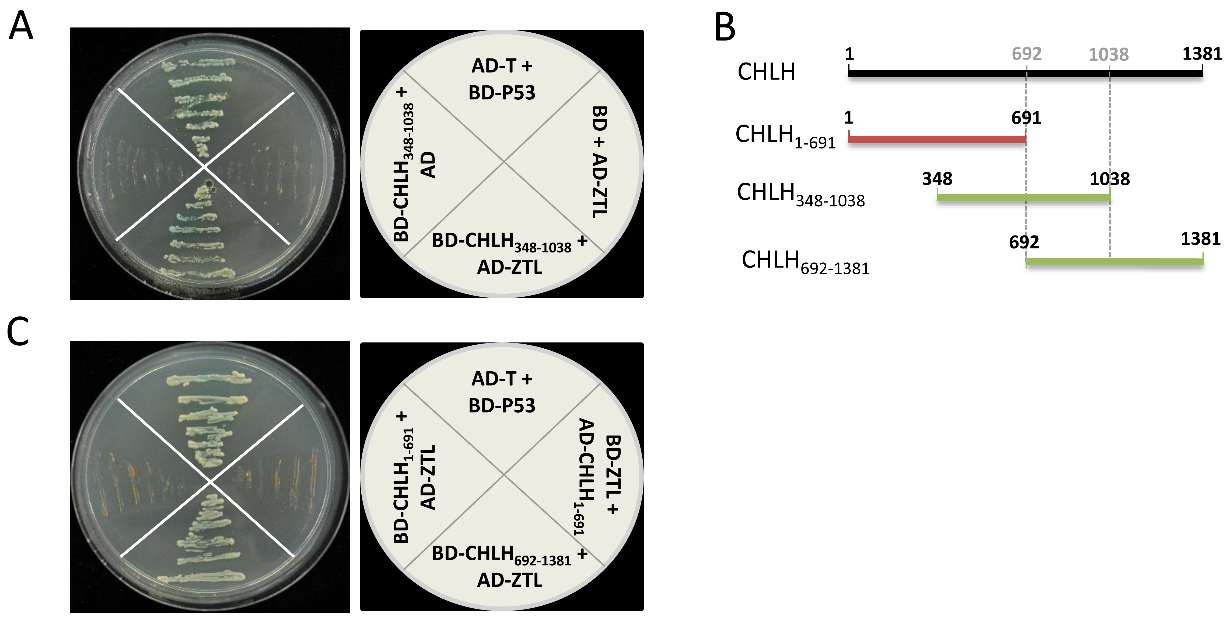
**

**Supplementary Figure S2**. **Detection of the interaction of the N-terminus and central region of CHLH with ZTL in the yeast two-hybrid assay.**

**(A)** Validation of the interaction between ZTL and the central region of CHLH_348-1038_ in the yeast cells. Yeast strain AH109 co-transformed with the BD-CHLH_348-1038_/AD-ZTL constructs was able to grow on SD4-. BD-P53/AD-T was taken as a positive control. BD-CHLH_348-1038_/AD and BD/AD-ZTL were taken as negative controls. The experiments were repeated three times with the same results.

**(B)** Summary of the interaction of the truncated CHLHs with the ZTL in the yeast two-hybrid system. The red line indicates no interaction, and the green lines indicate an interaction.

**(C)** Validation of the interaction between ZTL and the N-terminus of CHLH_1-691_ in the yeast cells. Yeast strain AH109 co-transformed with the BD-CHLH_1-691_/AD-ZTL and BD-ZTL/AD-CHLH_1-691_ constructs were not able to grow on SD4-. BD-P53/AD-T and BD-CHLH_692-1381_/AD-ZTL were taken as positive controls. The experiments were repeated three times with the same results.

**
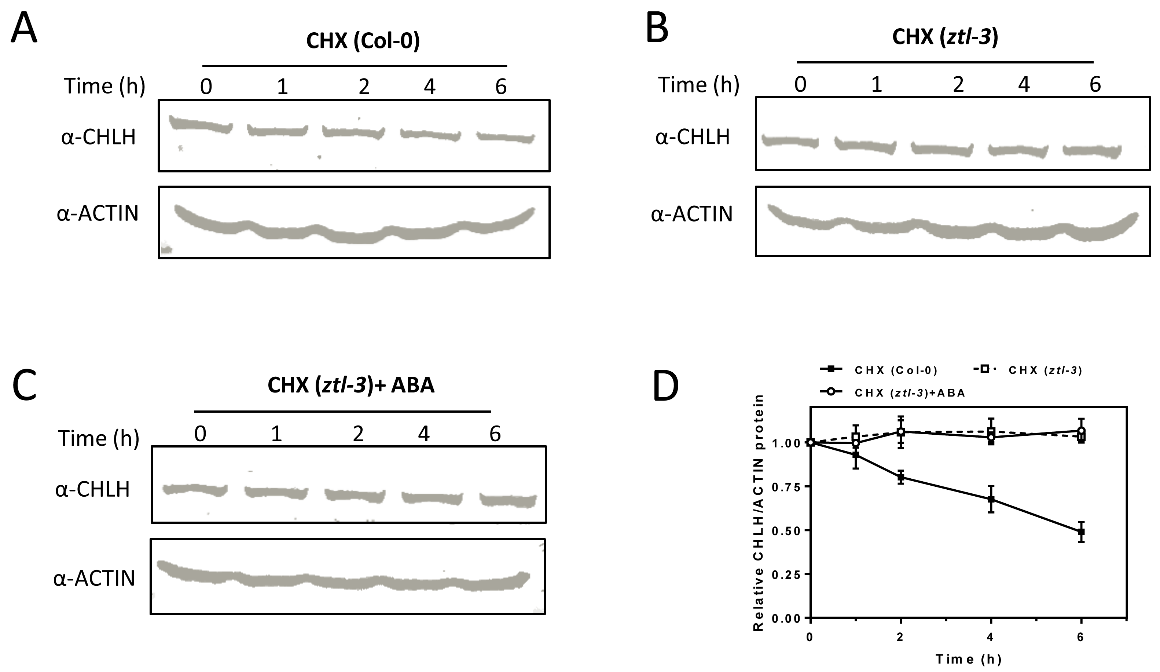
**

**Supplementary Figure S3**. **ABA does not induce CHLH proteasomal degradation in *ztl-3* mutant.**

**(A-C)** Immunodetection of CHLH protein in *in vivo*. Seedlings were grown in LgD, and seedlings were treated with Cycloheximide (CHX) for two hours at ZT3, then the seedling were treated with or without 100 µM (±)ABA.

**(D)** Quantification of CHLH protein abundance in **(A-C)**. Data are shown as means ± SE of three independent experiments relative to the initial value and normalized to ACTIN abundance.

**Supplemental Table 1.** Primers used for constructing vectors.

| **Generation of transgenic plants (*ZTL*)**  forward primer 5’- GGGGACAAGTTTGTACAAAAAAGCAGGCTTCATGGAGTGGGACAGTGGTTC -3’  reverse primer 5’- GGGGACCACTTTGTACAAGAAAGCTGGGTCCGTGAGATAGCTCGCTA -3’ |
| --- |
| **Generation of ZTL-pGADT7 plasmid**  forward primer 5’-GGAATTCCATATGATGGAGTGGGACAGTGGT-3’  reverse primer 5’-CCGCTCGAGTTACGTGAGATAGCTCGCTAGT-3’ |
| **Generation of ZTL-pGBKT7 plasmid**  forward primer 5’- GGAATTCCATATGATGGAGTGGGACAGTGGT -3’  reverse primer 5’- ACGCGTCGACTTACGTGAGATAGCTCGCTAG -3’ |
| **Generation of CHLH_1-691_-pGADT7 plasmid**  forward primer 5’- GACGTACCAGATTACGCTCATATGATGGCTTCGCTTGTGTATTCT -3’  reverse primer 5’- CTACGATTCATCTGCAGCTCGAGTTAGATAAGACTGTCGGGAAAAC -3’ |
| **Generation of protein purification plasmids**  (*ZTL*) forward primer 5’- CTGTACTTCCAATCCAATATGGAGTGGGACAGTGGTTC -3’  (*ZTL*) reverse primer 5’- CCGTTATCCACTTCCAATTTACGTGAGATAGCTCGCTA -3’  (*CHLH*) forward primer 5’- CTGTACTTCCAATCCAAT ATGGCTTCGCTTGTGTATTCTCCA-3’  (*CHLH*) reverse primer 5’- CCGTTATCCACTTCCAAT TTATCGATCGATCCCTTCGATCTT-3’ |
| **Generation of ZTL-cLUC plasmid**  forward primer 5’- CGGGGTACCATGGAGTGGGACAGTGGTTCC-3’  reverse primer 5’- ACGCGTCGACTTACGTGAGATAGCTCGCTA-3’ |
